# The challenge of secondary cardiovascular prevention in very high Lipoprotein (a) level: a case report

**DOI:** 10.1093/ehjcr/ytaf389

**Published:** 2025-08-09

**Authors:** Andrea D’Amuri, Pietro Di Gangi, Mauro Pagani, Corrado Lettieri

**Affiliations:** Medical Department, General Medicine Unit, Azienda Ospedaliera Carlo Poma, ASST Mantova, Str. Lago Paiolo 10, Mantova 46100, Italy; Cardio-Thoracic-Vascular Department, Cardiology Unit, Azienda Ospedaliera Carlo Poma, ASST Mantova, Str. Lago Paiolo 10, Mantova 46100, Italy; Medical Department, General Medicine Unit, Azienda Ospedaliera Carlo Poma, ASST Mantova, Str. Lago Paiolo 10, Mantova 46100, Italy; Cardio-Thoracic-Vascular Department, Cardiology Unit, Azienda Ospedaliera Carlo Poma, ASST Mantova, Str. Lago Paiolo 10, Mantova 46100, Italy

**Keywords:** Case report, Lipoprotein a, Dyslipidemia, Cardiovascular risk, Lipid-lowering therapy

## Abstract

**Background:**

While advances in technology and procedural techniques have significantly improved outcomes post-PCI, two pharmacological strategies have gained particular attention for their effectiveness in reducing long-term cardiovascular (CV) risk: anti-platelet therapies and lipid-lowering therapies (LLT). The 10-year recurrence risk for major CV events remains as high as 10–30%, due to various pathophysiological pathways collectively known as residual risk (RR), even with optimal CV risk factor management after acute coronary syndrome (ACS). RR includes factors such as elevated lipoprotein(a) [Lp(a)], triglycerides, pro-thrombotic states, hyperglycemia, and persistent subclinical arterial inflammation.

**Aims:**

This case highlights the challenge of managing a patient with multiple recurrent cardiac ischaemic events and in-stent restenosis, despite good medical therapy and no other significant CV risk factors except for markedly elevated Lp(a) levels.

**Conclusion:**

Three critical aspects of daily practice emerge from our observation. First, Lp(a) is a valuable parameter for CV risk stratification in primary prevention. Second, measurement of Lp(a) post-CV event may provide valuable information on the risk of ischaemic recurrence, influencing decisions regarding long-term dual anti-platelet therapy (DAPT). Finally, this case illustrates the importance of a multidisciplinary approach in managing patients with very high cardiovascular risk. Close collaboration between cardiologists and lipidologists facilitated the identification of a rare lipid disorder and the decision to pursue lipoprotein apheresis, an intensive but effective treatment option for lipid metabolism disorders lacking conventional medical therapy.

Learning points

- Elevated Lp(a) is under-recognized in CV risk stratification, particularly in secondary prevention settings, and current treatment guidelines inadequately address Lp(a)-related residual risk due to limited pharmacological options.
- Prolonged DAPT, PCSK9 inhibitors, and lipid apheresis are potential strategies to mitigate this risk until specific anti-Lp(a) therapies become available.

## Introduction

The risk of acute coronary syndrome (ACS) recurrence remains high after PCI without sustained management of cardiovascular (CV) risk factors. Two key strategies to reduce long-term CV risk are anti-platelet therapies and lipid-lowering therapies (LLT). Dual anti-platelet therapy (DAPT) is recommended for 12 months post-PCI, with extended DAPT (ASA and ticagrelor 60 mg twice daily) for high-risk patients without bleeding concerns.^1^ Aggressive LLT is essential, aiming for a low-density lipoprotein cholesterol (LDL-C) reduction of >50%, targeting <55 mg/dL in CV disease (CVD) patients, and <40 mg/dL for those with multiple CV events within 2 years.^2^ However, even with optimal CV risk factor management after ACS, the 10-year recurrence risk for major CV events remains as high as 10–30% due to ‘residual risk’ (RR) from elevated lipoprotein-a [Lp(a)], triglycerides, pro-thrombotic states, hyperglycemia, and subclinical arterial inflammation.^3^

This case report presents a patient with multiple recurrent CV events despite good treatment, primarily due to extremely high Lp(a), underscoring challenges in managing RR.

## Summary figure

**Figure:**
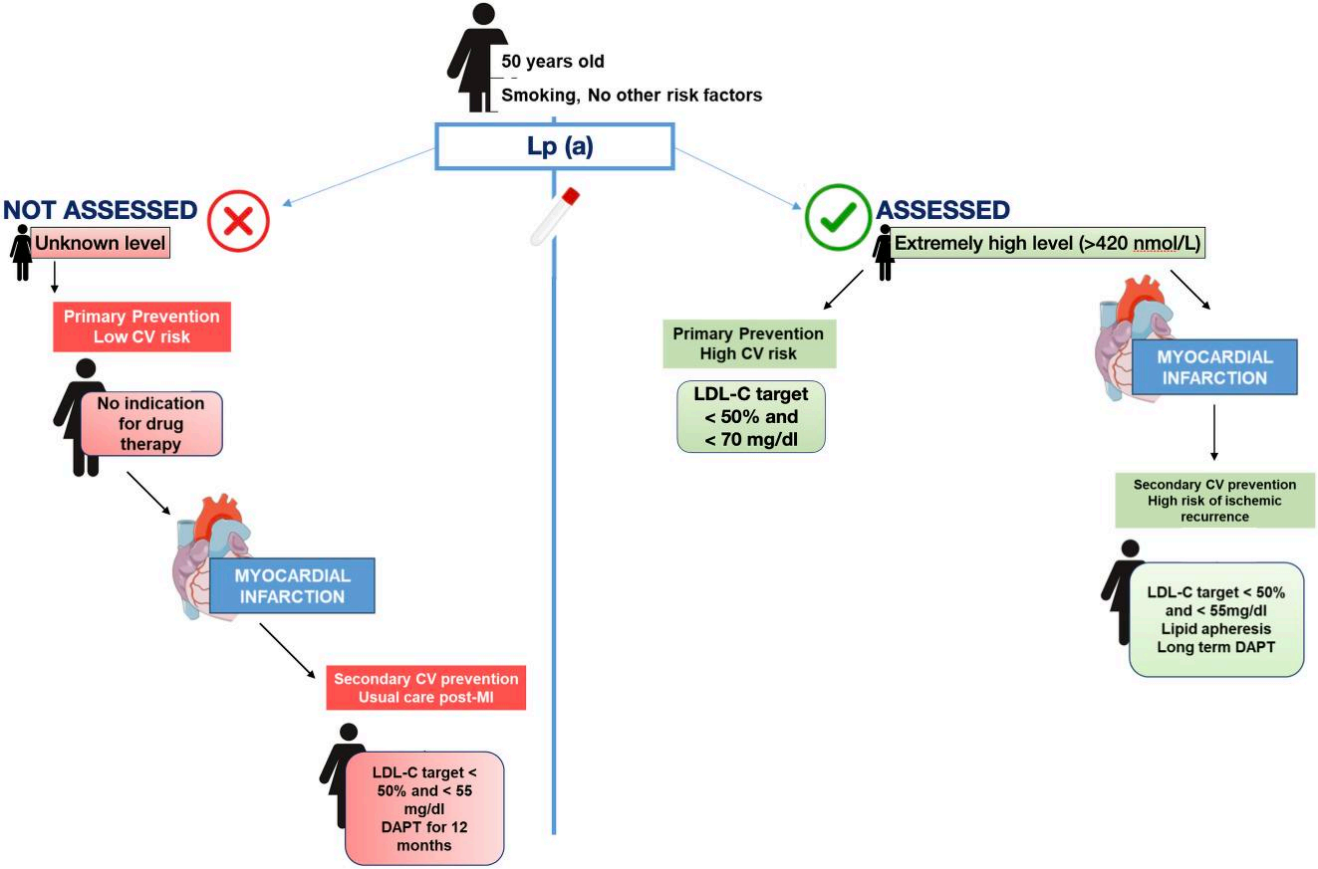


## Case presentation

The case is a female patient with no significant personal medical history. She was an active smoker (30 pack-years) and had a family history of CVD. She adheres to a healthy diet according to WHO recommendations and maintains an active lifestyle.

In 2010, at age 52, she was admitted for unstable angina and was found to have mild and diffuse coronary atherosclerosis with critical stenosis of the right coronary artery (RCA), treated with PCI and a drug-eluting stent (DES). During hospitalization, her lipid profile revealed a total cholesterol (TC) of 193 mg/dL, high-density lipoprotein cholesterol (HDL-C) of 61 mg/dL, triglycerides of 80 mg/dL, and LDL-C of 116 mg/dL. She was discharged with DAPT (ASA and clopidogrel) for 12 months and rosuvastatin 20 mg/day (*Table 1*).

**Table 1:** Timeline of the events

Year	Event/Intervention	Details
2010	Initial ACS Diagnosis	Patient (T.G.), 52, presented with unstable angina. PCI performed with a DES in the RCA. Lipid profile: LDL-C 116 mg/dL. Discharged on DAPT.
May 2023	Recurrent Angina	Patient presented with exertional angina. Intra-stent stenosis (90% proximal RCA, 70% mid RCA). PCI performed with two additional DES. LDL-C: 78 mg/dL.
Early September 2023	Non-ST Elevation MI (NSTEMI)	Diagnosed with 99% sub-occlusive intra-stent restenosis in RCA. Treated with a drug-coated balloon. LDL-C: 18 mg/dL (calculated), 39 mg/dL (direct).
Late September 2023	Lipidology Evaluation	Lp(a) measured >428 nmol/L. Incomplete corneal xanthoma observed. Prolonged high-potency DAPT (ASA + ticagrelor) and lipid apheresis recommended.
Ongoing	Multidisciplinary Management	Prolonged DAPT, PCSK9 inhibitors and lipid apheresis to manage high residual risk

ACS, Acute Coronary Syndrome; ASA, Acetyl Salicylic Acid; DAPT, Dual Anti-Platelet Therapy; DES, Drug Eluding Stent; LDL-C, Low-Density Lipoprotein Cholesterol; Lp(a), Lipoprotein (a); PCI, Percutaneous Coronary Intervention; PCSK9, Proprotein Convertase Subtilisin/Kexin type 9; RCA, Right Coronary Artery.

In May 2023, she was readmitted for exertional angina, showing a 90% intra-stent stenosis in the proximal RCA and a 70% stenosis in the middle RCA. Restenosis was pre-dilated with an Artimes 2.5 × 20 mm SC balloon, followed by two DES Frontier stents (3 × 38 mm, 3 × 12 mm) using the stent-in-stent technique. Post-dilation with an Apollo 3.5 × 15 mm balloon at 26 atm achieved excellent angiographic results and optimal stent expansion. At that time, her TC was 153 mg/dL, HDL-C 58 mg/dL, triglycerides 85 mg/dL, and LDL-C 78 mg/dL. She was discharged with DAPT (ASA and clopidogrel) for 12 months, rosuvastatin 20 mg/day, ezetimibe 10 mg/day, and alirocumab 150 mg twice a month.

In September 2023, the patient presented with non-ST elevation MI (NSTEMI). Imaging showed a 99% sub-occlusive intra-stent restenosis in the RCA, treated with a drug-coated balloon. During hospitalization, her TC was 97 mg/dL, HDL-C 67 mg/dL, triglycerides 59 mg/dL, and LDL-C was 18 mg/dL (calculated) and 39 mg/dL (direct measurement). She was discharged with high-potency DAPT (ASA and ticagrelor), with her LLT regimen unchanged, though a lipid clinic evaluation was recommended before discharge.

During the lipidology evaluation, an incomplete corneal xanthoma was observed, and further blood tests revealed an Lp(a) level above the calibration scale (>428 nmol/L). Following a consultation between her cardiologist and lipidologist, the patient was recommended for prolonged high-potency DAPT (ASA + Ticagrelor) and lipid apheresis.

## Discussion

This case highlights the challenge of managing a patient with recurrent cardiac ischaemic events and in-stent restenosis, despite good medical therapy and the absence of other significant CV risk factors, aside from markedly elevated Lp(a) levels. This case underscores several aspects critical to daily clinical practice.

First, Lp(a) serves as a valuable parameter for CV risk stratification in primary prevention. Before her first CV event, the patient would have been considered low-risk, meaning she might not have been identified for pharmacologic risk management. Since individual CV risk is continuously amplified by increasing Lp(a) levels, according to the 2019 EAS/ESC guidelines on dyslipidemia management^2^ and expert consensus,^4^ individuals with very high Lp(a) levels (>320 nmol/L) should be classified as high CV risk. For more moderate elevations, tailored LDL-C reduction targets are advised to counterbalance the additional risk conferred by elevated Lp(a). However, the 2021 ESC guidelines for CV prevention^5^ do not endorse the routine use of circulating biomarkers due to their limited ability to reclassify most individuals. Despite this, our case illustrates how extreme Lp(a) values can have clear clinical implications and be more straightforward for clinicians to interpret than moderate elevations. A key insight from this case is that Lp(a) measurement may also hold value following PCI. Current guidelines do not consider Lp(a) as a risk modifier in secondary prevention,^1,2,4,5^ yet evidence suggests that elevated Lp(a) levels contribute significantly to the RR.^2,4^ Excluding acute phases, Lp(a) levels are primarily determined by genetic background. Observational data support a link between Lp(a) and recurrent ischaemic events post-PCI, both due to atherosclerosis progression and in-stent restenosis.^6^ Measurement of Lp(a) post-CV event may provide valuable information on the risk of ischaemic recurrence, which could influence decisions regarding long-term DAPT. In fact, studies indicate that prolonged DAPT is associated with reduced recurrence of CV events in patients with elevated Lp(a) following PCI.^7–9^

The management of thrombotic RR is of particular importance in a context such as that of particularly high Lp(a) values as to date there are still no approved medical therapies for this lipid disorder.^4^

Several therapies targeting Lp(a) are currently in development. Antisense oligonucleotides, such as Pelacarsen, have demonstrated a 66–92% reduction in Lp(a) levels and are now being evaluated in phase 3 trials (NCT04023552). Small-interfering RNA (siRNA) therapies, including Olpasiran, Zerlasiran, and Lepodisiran, have achieved Lp(a) reductions exceeding 97%, with Olpasiran also undergoing phase 3 trials (NCT05581303). Muvalaplin, the first oral Lp(a) inhibitor, has shown promise but is in the early stages of development.^10^ All the Proprotein Convertase Subtilisin/Kexin type 9 inhibitors (PCSK9i), both monoclonal antibody (alirocumab and evolocumab) and siRNA (inclisiran), have shown to reduce Lp(a) by ∼20%.^11^ Despite this effect, current guidelines suggests to consider the use of PCSK9i in secondary prevention based on the LDL-C parameter alone.^2^ Currently, the only approved treatment to reduce Lp(a) levels is lipid apheresis (LA). LA allows extracorporeal lipoprotein separation and lowers LDL-C and Lp(a) by over 70%, with observational studies suggesting a reduced incidence of CV events.^12^ A randomized trial (NCT02791802) is underway to confirm its efficacy. However, LA is limited by high costs, frequent lengthy sessions, and restricted availability. Nevertheless, LA is approved in several countries and in Italy it is indicated for individuals with Lp(a) > 60 mg/dL (∼150 nmol/L) and progressive coronary artery disease despite optimal medical therapy.^13^

This case highlights the value of a multidisciplinary approach in high CV-risk patients, where cardiologists and lipidologists collaborated to diagnose a rare lipid disorder. Such integration aids in managing complex cases, supports unconventional therapies like LA or long-term DAPT, and helps detect hereditary lipid disorders. High Lp(a) levels suggest the need for cascade screening to identify at-risk relatives for early preventive treatment. Our patient was questioned about this, and she has no siblings, no children, and no contact with second-degree relatives.

This report has some limitations. Firstly, there was a lack of intensification of LLT between the patient’s first two CV events, a well-documented issue in real-life.^14^ However, while this omission is associated with a 12–15% increased risk of recurrence,^14^ an Lp(a) increase like the one observed in our patient is estimated to confer a 300–400% higher risk.^15^ Moreover, DAPT benefits in high Lp(a) patients lack strong evidence, with no dedicated trials. However, such extreme Lp(a) levels are rare (<1% prevalence) limiting research. The 2023 ESC ACS guidelines (Class IIa-A) support long-term DAPT for high ischaemic and low bleeding risk patients such the case profile. We believe that this case highlights the importance of clinician judgment in tailoring secondary CV prevention and applying guideline-based long-term DAPT.

## Conclusion

This case highlights the importance of Lp(a) in CV risk stratification and management, particularly in recurrent CV events despite good therapy. Prolonged DAPT, PCSK9i and lipid apheresis could benefit patients with extremely high Lp(a). A multidisciplinary approach remains critical in managing complex cases with substantial residual risk

## Patient perspective

After a multidisciplinary discussion, we proposed the patient to initiate lipid apheresis (LA). Informed about the procedure and the related benefits outweighing the risks, the patient declined the treatment. As a passionate traveler, she was worried about the detrimental impact of LA on her quality of life. Conversely, when we discuss about the option of long-term DAPT, along with its potential benefits and risks, the patient agreed with this therapy as a ‘bridging approach’ while awaiting the potential availability of specific Lp(a)-targeting drugs.

## Lead author biography


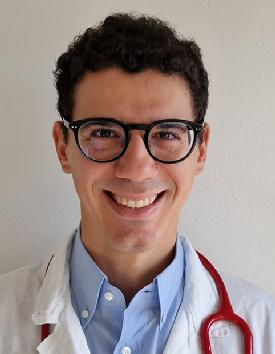


Dr Andrea D’Amuri is a specialist in Internal Medicine, currently serving at ASST Mantova’s Carlo Poma Hospital, where they lead the Lipid Clinic within the General Medicine Unit. With a strong academic foundation, they graduated with honors from the University of Ferrara, focusing on exercise and metabolic health. They specialized in managing cardiovascular risk, including lipid disorders, hypertension, diabetes, obesity, and metabolic syndrome, emphasizing nutrition and cardioprotective therapies. Previously, they worked at Ferrara University Hospital, gaining expertise in obesity, diabetes, and atherosclerosis. A dedicated researcher and practitioner, they also completed advanced lipidology training throughthe SISA Lipid Academy.

## Data Availability

The authors make the original data of the presented clinical case available upon appropriate request.

## Notes

**Consent:** The patient has given consent for the publication of the clinical case according to COPE guidelines.
